# The antioxidant potential of bacoside and its derivatives in Alzheimer's disease: The molecular mechanistic paths and therapeutic prospects

**DOI:** 10.1016/j.toxrep.2025.101945

**Published:** 2025-02-04

**Authors:** Dipanjan Karati, Swarupananda Mukherjee, Souvik Roy

**Affiliations:** a Department of Pharmaceutical Technology, School of Pharmacy, Techno India University, Kolkata, West Bengal 700091, India; b Department of Pharmaceutical Technology, NSHM Knowledge Campus, Group of Institutions, Kolkata, 124 B.L. Saha Road, Kolkata, West Bengal 700053, India

**Keywords:** Bacoside, Neuroprotective effect, Superoxide dismutase, β-Amyloid, Alzheimer disease, Natural compounds

## Abstract

Central nervous system disorders are likely to have a substantial effect on the worldwide healthcare demands of humanity in this era. Alzheimer’s disease (AD) is a senile decay of neurons. Extracellular beta-amyloid accumulation and intracellular tau hyperphosphorylation are two key characteristics of the pathogenesis of AD. Because of the multifactorial character of many disorders, new medicine-based psychoactive treatments have had limited success. As a result, there is a growing demand for innovative products that can target different receptors and improve behavioral abilities on their own or in combination with established treatments. In recent years, both industrialized and developing countries have seen a surge in herbal products based on traditional knowledge. According to recent research, bacoside and its congeners can dramatically lower the build-up of amyloid-β plaques, which are a defining feature of AD. This decrease is explained by bacoside's capacity to regulate β-secretase activity, which lowers the production of amyloid-β. Ayurveda is a medical science that focuses on the use of naturally occurring plant products to treat ailments. Many neuroprotective plants are said to be found in Ayurveda. The key physiological dysfunctions linked to tau aggregates, which contribute to dementia and behavioral inconsistencies, include the formation of reactive oxygen species, augmented neuronal swelling, and neurotoxicity. Here, we have focused on bacopa as an anti-Alzheimer medication. Bacoside A, Baccoside B, Apigenin, Betullinic acid, etc. are the pharmacologically active congeners of Brahmi belonging to several chemical families. In this review, the neuroprotective properties, pharmacological effectiveness, and molecular mechanism of bacoside scaffolds against AD have been discussed.

## Highlights

• Bacopa active against neuroinflammation through NF-κB signaling cascade and MAPKs.
• Pharmacological effectiveness and molecular mechanism of bacoside scaffolds against AD.
• Bacopa monnieri extracts reduce tau hyperphosphorylation.
• *In-silico* study regarding bacoside as potential congener against AD.
• Brahmi affects neuronal and glial plasticity.

## Graphical Abstract

**Figure:**
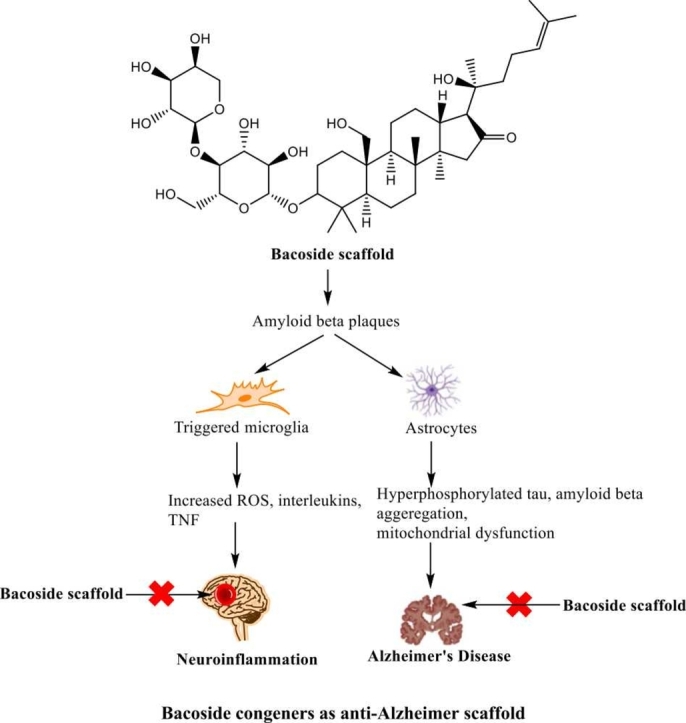
Bacoside congeners as anti-Alzheimer scaffold

## 1 Introduction

Alzheimer's disease (AD) is a terrible, irreversible neurological ailment marked by memory loss, disorientation, and augmented confusion [1]. The development of intracellular neurofibrillary tangles, responsive microgliosis, and astrogliosis, as well as the formation of extracellular amyloid-beta (Aβ) deposits in senile plaques, are the main histological features of AD [2]. The involvement of the middle temporal lobe and the hippocampus, which control recent memory, causes early impairment of recent memory [3]. AD affects brain cells, resulting in a reduction in cognitive function. The domino consequences of AD include senile dementia, intra-neuronal neurofibrillary tangle development, and beta-amyloid protein deposition in the form of amyloid plaques in the cerebral parenchyma. It is primarily caused by a clumping of aberrant amyloid beta protein, hyperphosphorylation of tau protein, and cholinergic system dysfunction, among other things [4], [5], [6], [7], [8], [9]. Other parts of the brain may become involved, resulting in sleep disorders, judgment problems, psychological abnormalities, and pyramidal and extrapyramidal movement symptoms [9], [10], [11], [12], [13], [14], [15]. Due to their superior biosafety profile than synthetic pharmaceuticals, herbal medications are garnering universal acceptance in more than 80 percent of the world's population. Numerous research documents stated the therapeutic potential of *Bacopa monnieri* (Brahmi) in several diseases [16], [17], [18], [19], [20], [21]. The aforementioned therapeutic effects of alcoholic or aqueous extracts of bacopa/bacoside A in the CNS are brought about by the molecules crossing the BBB is attributed partly to the lipophilic nature of bacoside A and its derivative ebelin lactone [22]. We researched the literature extensively in this post on Bacopa, a nootropic herb with antioxidant, acetyl choline mimetic, anti-beta, amyloid, and decent protection profiles, which has the potential to aid in the treatment of AD. Findings on pharmacological or nonpharmacological methods to decrease the course of illness are crucial because there are currently no viable disease-modifying medications. Additionally, the necessity for research into early AD diagnosis has been underlined by the failure of promising medications in human clinical trials. Since AD's clinical symptoms are already accompanied by brain shrinkage and synaptic and neuronal loss, current therapies aimed at slowing the disease's progression are more likely to be successful before AD symptoms manifest, ideally at the earliest preclinical stage. The evaluation of alternative therapies, such as nutraceuticals, has resulted from the absence of efficacious AD medications and treatments. Numerous antioxidants, for instance, may improve cognitive function [23], [24], [25]. Nutraceuticals affect signalling pathways, which has an impact on a variety of neurodegenerative illnesses [26]. Nutraceuticals are nutritional supplements, herbal remedies, and nutrients that can improve one's quality of life, fight various illnesses, and preserve physical wellness. Bacosides derived from BM have anti-inflammatory, antioxidant, and Aβ aggregation inhibitor characteristics, making them promising therapeutic agents for AD. Current clinical research and scientific data supporting the therapeutic potential of BM extracts (BME), such as bacosides, in AD are presented in this review.

## 2 Inheritance of AD

### 2.1 Genes involved in AD

Several genes are involved in the etiology of AD. The utmost imperative, as well as calculated examples, are presenilin 1 (PSEN-1), amyloid precursor protein (APP), presenilin-2 (PSEN-2), and apolipoprotein E (APOE). APP and PSEN-1 gene transmutations consequence in primary onset AD; APOE mutation is executed in delayed-onset Alzheimer, while PSEN-2 has an added inconstant onset [27]. The PSEN-1 gene is found on chromosome 14q and affects intracellular Ca^2+^ signalling, membrane protein trafficking, and β-catenin stabilization. As a result of dismemberment at dissimilar places by γ -secretase, any mutation in the PSEN-1 gene outcomes in the synthesis of variable lengths of Aβ peptides. These peptides are strongly fibrillogenic, causing enhanced Aβ plaque aggregation in the brain [28]. The APP gene is found on chromosome 21q and produces a protein known as APP. α-, β-, and γ-secretases help to break down the peptide bonds of this protein. When the APP is cleaved by the β and γ-secretase enzymes, it generates a protein that can gather and form plaques in the brain, causing neural deterioration [29].

The PSEN-2 gene on chromosome 1q enciphers the PSEN-2 transmembrane protein, which has two isoforms, one of which is present in the brain. PSEN-2 mutations result in the build-up of A42 protein, which is a characteristic of AD [30]. The APOE gene is linked to the creation of Apo-E, a protein that aids in the elimination of A protein and protects neurons from the damage they cause. Apo-E protein comes in three distinct varieties: ε2, ε3, and ε4. According to studies, the mutant oxidized version of ε4 binds quickly, binds to Aβ protein, and forms amyloid plaques. It is also discovered that cholinomimetic action in the central nervous system has decreased [31]. As a result, Apo-E isoform ε4 is involved in the etiology of AD.

### 2.2 Oxidative stress (OS) in AD

OS plays a role in the buildup of tau protein, which has been hyperphosphorylated in AD neurofibrillary tangles, and Aβ plaques. This leads to synapse dysfunction, neuronal death, and cognitive impairment. OS may also impair the blood-brain barrier (BBB), which makes neuronal damage worse [32].

### 2.3 Apoptosis

The highly functioning and regulated process of apoptosis is believed to be the cells' deliberate death for a variety of reasons. By cleaving specific proteins in the cytoplasm and nucleus, caspases cause apoptosis inside the endoplasmic reticulum (ER), mitochondria, and lysosomes [50]. Apoptosis aids in the removal of careless cells from our body's processes, but when used improperly, it can have detrimental effects, as shown in AD. The in vitro and in vivo cell cultures indicate a link between AD and apoptosis. By cleaving certain proteins in the cytoplasm and nucleus, caspases can be triggered in response to apoptotic signals. They are thought to have a crucial role in how apoptotic processes are carried out. The development of Aβ plaques is mediated by these same caspases. GSK-3 plays two roles in apoptosis-related pathways: it promotes apoptosis in the intrinsic pathway and inhibits it in the extrinsic pathway. Many neurodegenerative diseases are mostly caused by excessive cell death, and a number of diseases, such as cancer, autoimmune diseases, and caspase-8 deficiency, are characterized by dysregulation of apoptotic signals. It is crucial to comprehend how the apoptosis pathways are structured [33].

### 2.4 Autophagy

An important cellular mechanism for preserving neural homeostasis, autophagy is also linked to the etiology of AD. In order to remove damaged organelles and misfolded proteins, such as tau and amyloid-beta (Aβ), which are characteristics of AD, it entails the breakdown and recycling of cellular components through lysosomes. These toxic aggregates can build up as a result of autophagy dysregulation in AD, aggravating neuronal dysfunction and cell death. Because of decreased autophagic flux, which disrupts the fusion of autophagosomes with lysosomes, autophagosomes—vesicles that sequester harmful materials—frequently accumulate in AD brains. In preclinical models, increasing autophagy via pharmacological or genetic treatments has demonstrated promise by encouraging the removal of tau and Aβ, making it a possible therapeutic target for slowing the course of AD [34].

## 3 Chemistry of bacoside congeners

**Fig. 1:**
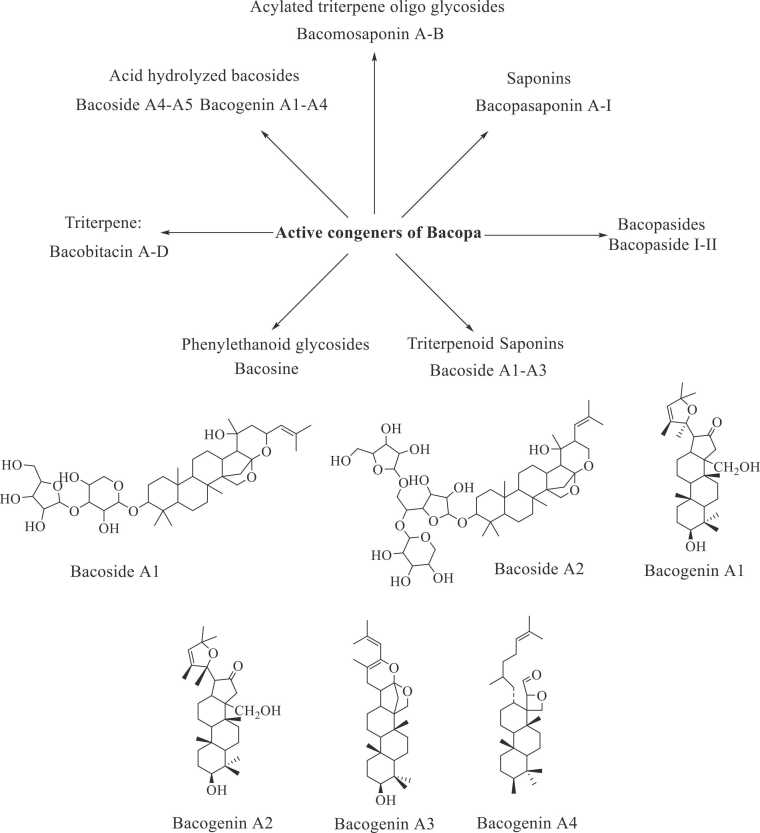
The phytoconstituents of bacopa.

Being one of the most common species, *B. monnieri* is a non-aromatic plant. It's a popular aquarium plant because of its capacity to grow in water. It is a little perennial creeping herb that belongs to the Scrophulariaceae family, which has 220 genera [35], [36], [37]. Three primary saponins found in this plant are bacoside A, bacoside B, and monnierin (Fig. 1). These chemical constituents are the dammarane category triterpenoid saponins. Apart from these compounds, Apigenin, Brahmine, and Asiaticoside, which are flavonoids, alkaloids, and glycosides, are also present. Saponins are the most potent pharmacologically efficient of these biologically active components. Bacoside-A and bacopaside I account for more than 96 % of Brahmi's total saponins by weight [38], [39], [40], [41], [42], [43], [44], [45].

## 4 Neuroinflammation in AD

One of the most essential pathogenic characteristics of AD is neuroinflammation. Several events, such as traumatic brain damage, exposure to chemicals, and protein build-up, set off the inflammatory reactions of the central nervous system (CNS), which are facilitated by the CNS's resident macrophages, called microglia. The primary CNS modulators of neuroinflammation are microglia. Microglia preserves the CNS's regular function and resting state in a steady microenvironment. Microglia can transition from quiescent to activate in response to stimuli that alter the microenvironment [46]. Pro-inflammatory proteins like interleukin 1 beta (IL-1β), IL-6, and TNFα are produced in greater amounts by this activated microglia, which results in prolonged neuroinflammation and important processes linked to neurodegenerative diseases. These processes include loss of synaptic function, decline in cognition and memory, mitochondrial damage, and direct or indirect induction of tau and Aβ protein clustering [47]. In activated microglia and astrocytes, glial inflammatory activation is mediated by several mechanisms, including the NF-κB signaling cascade and MAPKs. Neurodegenerative illnesses are brought on by dysregulation of the NF-κB and MAPK signaling cascade, which impacts the brain [48], [49], [50]. Being a well-known herb in the ayurvedic medical system, BME's impact on downregulating neuroinflammation has been studied illustratively. It was shown that monocytes activated with LPS produced considerably less IL-6 and TNF-α when fractions of BME were present [51]. BM has been subjected to new scenarios in which suppressing pro-inflammatory cytokines may ultimately result in inflammation and trigger the programmed cell death pathway. The cysteine aspartate proteases, or caspases, have a role in both inflammatory and apoptotic processes. According to reports, Brahmi inhibits the activity of the enzymes caspase 1 and 3, MMP-3, which govern systemic inflammation in cells [52]. Moreover, it attenuates the innate immune system's pro-inflammatory cytokines [53]. Bacopa contains bioactive chemicals shown in several studies to prevent various neurological problems. Bacopa is also a powerful antioxidant and anti-inflammatory herb.

## 5 Tau pathology

Aggregation of tau and Aβ peptides are key clinical characteristics of a class of neurodegenerative disorders. Neurons are poisoned by these protein aceous clumps. Microtubule stability depends on Tau, a microtubule-associated protein (MAP). Research has shown that tau protein hyperphosphorylation results in tauopathies, a significant defining feature of several neurodegenerative illnesses in humans, including Parkinson's and Alzheimer's diseases. [54], [55]. Because of its reduced affinity for microtubules, hyperphosphorylated tau protein destabilizes the cytoskeleton and reduces axonal transport. Tauopathy, which is caused by hyperphosphorylated tau protein, encourages the buildup of misfolded tau protein into neurofibrillary tangles (NFT), which impairs neurons and causes cell death. [56], [57]. According to recent research, plant-derived polyphenols prevent tau aggregation and may be a good option for AD treatments. The viability percentage of PC12 cells devoid of NGF was used to assess BM's capacity as a tauop athy protectant. According to their research, BME may decrease both the total expression of tau protein and the phosphorylation of tau protein at the tau-1 site in PC12 cells that are not receiving NGF. Activating the PI3K/Akt signaling, which is governed by a number of intricate regulatory mechanisms, including its involvement in the pathophysiology of AD, may accomplish this. Reduced tau phosphorylation and GSK-3β activity are linked to increased PI3K activity [58].

## 6 Pharmacological preface of bacoside in AD

The medicinal herb Bacopa monnieri has long been used in traditional Ayurvedic medicine. It is widely documented to affect neuropharmacology and cognitive function potentially [59], [60]. The possibility that bacoside might improve cognitive function—specifically, learning, memory, and attention—has been studied. It is believed that some neurotransmitters associated with cognitive processes, including acetylcholine, serotonin, and GABA (gamma-aminobutyric acid), are more highly active [61]. Studies have shown that bacoside and its scaffolds have neuroprotective qualities, which means they can aid in survival and shield brain cells from damage. It may mitigate or even stop the loss of neurons caused by diseases like AD [62]. Persistent inflammation in the brain can worsen neurodegenerative disorders. Bacopa monnieri's anti-inflammatory properties have been found to reduce brain inflammation and may reduce the risk of neurodegenerative disorders [63].

**Fig. 2:**
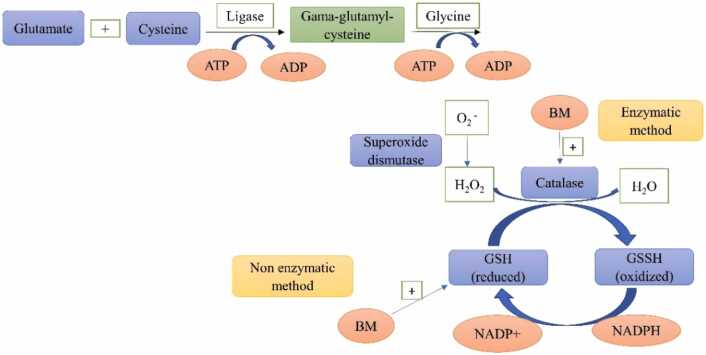
Enzymatic and non-enzymatic mechanisms of Brahmi.

Bacoside A and B are two main chemicals (saponins in nature) present in Brahmi. The major component responsible for the memory-enhancing action is bacoside A. Bacosides A and B have different optical rotations, with Bacoside A having a levorotatory structure and Bacoside B having a dextrorotatory structure. This plant's components have been linked to a variety of pharmacological effects. Brahmi's chemical components have vital biological roles in cancer, brain illness, cardiac disease, gastrointestinal disease, and blood sugar levels. The most commonly recommended drugs for AD include donepezil, rivastigmine, galantamine, and memantine. However, only approximately 20 % of AD patients respond moderately to these drugs, with benefits lasting on average six to twelve months and sometimes severe side effects. As a result, more effective pharmacological treatments with fewer side effects need to be developed and tested as soon as possible. Bacoside A and Bacoside B are compounds found in the plant BM, which are used as herbs in Ayurvedic medicine and may be utilized to treat Alzheimer's disease [64], [65]. The brain is principally vulnerable to free radical impairment because of unsaturated fatty acids in the cell membrane, a greater metabolic rate, decreased activity of antioxidants such as catalase (CAT) and glutathione peroxidase (GPx), and the cytotoxic effects of glutamate. The anatomy has a variety of free radical scavenging mechanisms, both enzymatic and non-enzymatic. Enzymes such as catalase, superoxide dismutase (SOD), and glutathione reductase are the first line of defence against ROS. Non-enzymatic antioxidants such as selenium, vitamins A, E, and C, glutathione (GSH), and coenzyme Q10 protect neural tissue against free radical damage. The disproportion between the defensive antioxidant mechanistic pathway and free radical scaffolds, on the other hand, is critical for free radical damage in the elderly, which leads to cognitive decline and aging. The action of BM extract on increased concentrations of Glutathione (GSH) and different enzymatic antioxidants including CAT, GPx, and SOD, as well as a free radical scavenging agent, is the main role of BM extract as an antioxidant (Fig. 2). As a result, some doses of Brahmi may be used to treat AD-related memory and cognitive impairment [66], [67], [68].

## 7 Effects of BM on major neurotransmitter systems involved in AD

### 7.1 Cholinergic system

Attention, recalling capacity are all aided by the neurotransmitter acetylcholine (ACh), which is held in vesicles in the terminal regions of neurons and released in response to an action potential [69]. The choline acetyltransferase enzyme (ChAT) catalyzes the conversion of acetyl-CoA and choline into ACh in presynaptic terminals [70]. In a neuropathological review of bacopa, the idea of replenishing ACh through choline acetyltransferase stimulation (synthesis of ACh) rather than AChE inhibition was suggested [71]. AChE enzyme helps to end ACh neurotransmission in a variety of cholinergic pathways. As a result, decreased AChE activity leads to augmented ACh amounts in the brain, enlightening learning and recalling capabilities. In a study evaluating the neuroprotective advantages of BM in an animal model of dementia, Saini et al. looked at the activity of membrane-bound enzymes (Na+, K+, and ATPase), and showed that BM supplementation restored their function, however AChE activity decreased, which could be associated to its cognitive enhancing effect [72].

### 7.2 Glutaminergic system

Glutamate, a neurotransmitter, which has an excitative action on the brain, helps to maintain learning and memory capabilities. Glutamatergic neurotransmission in the hippocampal formation is a biological underpinning for recalling capabilities of the brain. An experiment on subchronic phencyclidine-administered rats with abnormalities in new object identification found that cognitive deficiencies might be recovered with bacosides therapy. Utmost significantly, the immunohistochemistry research in this schizophrenic rat model proposed the amplified appearance of VGluT3 (vesicular glutamate transporter 3), VGluT1 (Vesicular glutamate transporter 1) and VGluT2 (vesicular glutamate transporter 2), density in the prefrontal cortex, striatum and CA1–3 of the hippocampus. The neurotransmitter glutamate can be transported into synaptic vesicles before being released into the synaptic cleft by this transporter protein. The regulating function of bacopa on metabotropic glutamate-8 receptor (mGluR8) and NMDA receptor 1 (NMDAR1) gene expression was revealed in a prior study on pilocarpine-tempted temporal cortex epilepsy in rats. Because hippocampus formation is essential for procedural memories, BM's repair of these receptors is the key to restoring pilocarpine-impaired declarative memory [73].

## 8 Brahmi on neuronal and glial plasticity

The ability of the brain to adjust to stress, known as neuronal or glial plasticity, involves complicated processes such as the reorganization of synaptic connections and the network of neurons [74]. A member of the neurotrophin family, brain-derived neurotrophic factor (BDNF), is an imperative pointer of neural plasticity. It correspondingly has an important effect in the transcription of the gene Arc, which is linked to neural pliability and reminiscence. A person with ignoble amounts of BDNF is predisposed to AD [75]. Glial fibrillary acidic protein (GFAP) is a crucial pointer of glial elasticity that influences astrocyte morphology, neuroglia connections, and memory formation pathways [76]. In amnesiac situations, GFAP expression is also significantly reduced. In their work, Konar et al. [77] found that when mice were given scopolamine, the expression of BNDF, Arc, and GFAP was considerably reduced. They discovered that giving BM alone boosted BDNF and Arc expression by 1.3 and 2 times, respectively, but did not increase GFAP expression. Pre-and post-administration of BM to scopolamine-treated mice increased the expression of plasticity markers in the cerebrum, with the impact being more pronounced in the expression of BDNF and Arc than GFAP. As a result, BM plays a key role in improving brain plasticity through several methods.

## 9 Effect of BM on lessening of β-amyloid

AD's most significant pathophysiological mechanism is the deposition of Aβ protein in the brain parenchyma [78], [79]. Methanolic BME almost completely prevented the development of amyloid fibrils and significantly separated the pre-formed amyloid fibrils, according to research by Mathew and his co researchers [80] on the anti-amyloidogenic potentials of several herbs. The treatment of BME in animals expressing APP and PSEN-1 mutation lowered the amounts of amyloidogenic proteins Aβ40 and Aβ42 in the brain by around 60 %, as shown by Holcomb et al.'s study [81] on PSAPP mice. This indicates BME's potential as a therapeutic approach in AD. Aβ protein-induced cell death in primary cortical culture was counteracted by BME, as shown by Limpeanchob et al. [82]. The quantity of acetylcholine esterase (AChE), a marker of neuronal injury, increased twofold in neurons treated with Aβ protein, but neurons treated with Aβ protein and Brahmi had almost normal AChE concentrations. Additionally, BME was found to have enhanced cell viability, decreased reactive oxygen species (ROS) in the cell, and possessed inherent antioxidant activity in the same research. This study demonstrated many BME Anti-AD pathways. Additional investigation in the form of human clinical trials is required to demonstrate its efficacy in AD.

The overall cellular mechanisms of Bacoside in AD have been noted in Table 1.

**Table 1:** Cellular mechanisms of Bacoside in AD.

Mechanism	Study highlights	References
AChE activity.	Inhibitory effect on amyloid peptide-activated intracellular AChE activity.	[83]
BDNF receptor expression	Up-regulation of BDNF receptor expression.	[84]
β-amyloid	Prevent self-assembly of oligomers.	[85]
Notch signaling pathway	Induced cell death and apoptosis.	[86]
Dopamine neurotransmitter	By maintain dopamine concentrations either by increasing dopamine synthesis or by inhibiting dopamine degradation.	[87]
GABA neurotransmitter	By restoration of GABAergic neurons.	[88]
ATPase system	Inhibition of calcium-ion influx into cell membranes.	[89]

## 10 *In silico* research regarding BM as potential scaffold against AD

Plant-based natural compounds have more potential pharmacological effects and are crucial in filling treatment gaps in a range of human disorders [90]. Natural compounds made from plants are already regarded in society as memory and learning enhancers. Bacopa is one of several medicinal herbs that help reduce the symptoms of AD and memory loss by initiating and blocking different chemical events at the molecular and cellular levels [91]. Numerous *in silico* studies have been conducted, revealing that bacoside and its scaffolds can bind many proteins and exhibit anti-Alzheimer's action.

According to Shoukat S. et al., bacopaside X has a binding energy of −17.87 kcal/mol and an affinity for the 3ZLT protein [92].

The proteins tau protein kinase I, or TPK I, and caspase-3, or CASP-3 (PDB IDs: 3KJF), are thought to be the causes of Alzheimer's symptoms. Roy S. and associates carried out the molecular docking [93]. According to the investigation, Bacopasaponin G had the best binding energy, measuring −9.6 Kcal/mol.

**Fig. 3:**
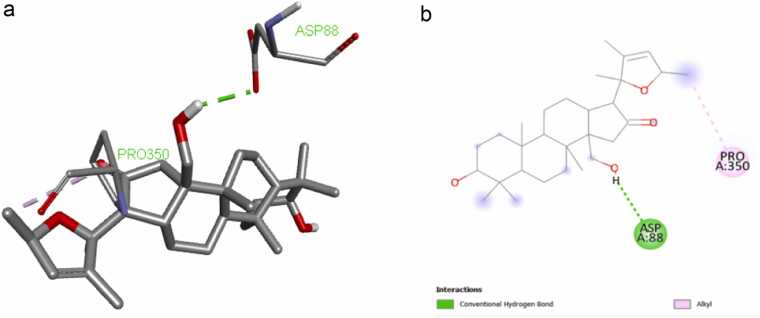
a. 3d interaction of bacogenin A1 and 4ZTH Fig. 3b. 2d interaction of bacogenin A1 and 4ZTH.

We have conducted *in silico* research to determine the efficacy of bacoside congeners against neuroinflammation associated with AD based on several docking studies pertaining to bacosides. In order to generate the congeners of these medicinal compounds in the future, an in-silico docking study of this molecule was conducted against MAPK 4ZTH. The binding energy was −8.6 kcal/mol. Figs. 3a and 3b (grid box x = 78, y = 46, z = 62) showed the interaction mode.

## 11 Conclusion and future prospects

Numerous traditional plants, like *B. monnieri*, have complex chemical combinations that have a range of biological and pharmacological properties. They have been utilized for anti-AD and as traditional remedies. The long-held theory states that plant components contain a variety of neuroprotective processes that enable them to be utilized as part of our well-being and are able to preserve the body's basic vitality. Brahmi is a nootropic plant that has been shown to boost cell metabolism by lowering a variety of risk factors. In this review paper, the neuroprotective efficacy, as well as potential applications of Brahmi in AD, is discussed. However, several drawbacks have been identified in several research works. Brahmi extract's effect on tauopathies has yet to be studied. Here, we have discussed in detail about the pharmacological activities of bacoside congeners on neuroinflammation, the effect on several neurotransmitter systems related to AD and the mechanism of action in preventing tau aggregation by Glutathione peroxidase enzymatic mechanisms. To create clinically useful medications, assessing each active molecule's bioavailability, toxicity, and structure-function connection is necessary. Because of its wide range of pharmacological activities, the phytochemistry region of BM must be further investigated and defined to have a comprehensive grasp of the role connected with each component of BME. More *in-silico*-based research on these bioactive phytoconstituents—bacoside A3, bacopaside I and II, bacopasaponin C, stigmasterol, betulinic acid, bacosine, luteolin, and monnieraside I–III—is also required to expedite the process of identifying crucial drug targets connected to neurological disorders. To effectively treat neurodegenerative illnesses, strategic medication development for the synergistic effects of BME's many elements will require clinical trials and more study.

## Abbreviation

Alzheimer disease (AD); amyloid-beta (Aβ); presenilin 1 (PSEN-1); amyloid precursor protein (APP); presenelin-2 (PSEN-2); apolipoprotein E (APOE); Bacopa monnieri extracts (BME); central nervous system (CNS); interleukin 1 beta (IL-1β); catalase (CAT); glutathione peroxidase (GPx); superoxide dismutase (SOD); choline acetyltransferase enzyme (ChAT); acetylcholine (ACh); glutathione (GSH); brain-derived neurotrophic factor (BDNF).

## Funding statement

Not applicable

## Ethics approval

Not applicable

## Author contribution statement

We declare that this work was done by the authors named in this article: S.R. and S.W.M. conceived and designed the study. D.K. and R.K. wrote the paper. D.K. performed the *in-silico* research and prepared the figures. D.K. prepared the figures. S.R. and S.W.M. drafted the manuscript. All authors have read and approved the final manuscript.

## CRediT authorship contribution statement

**Roy Souvik:** Conceptualization. **Karati Dipanjan:** Writing – original draft. **Mukherjee Swarupananda:** Conceptualization.

## Declaration of Competing Interest

The authors declare that they have no known competing financial interests or personal relationships that could have appeared to influence the work reported in this paper.

## Data Availability

No data was used for the research described in the article.

## References

[1] Sorour S.E., Abd El-Mageed A.A., Albarrak K.M., Alnaim A.K., Wafa A.A., El-Shafeiy E. (2024). Classification of Alzheimer’s disease using MRI data based on deep learning techniques. J. King Saud. Univ. -Comput. Inf. Sci..

[2] Heneka M.T., O’Banion M.K. (2007). Inflammatory processes in Alzheimer’s disease. J. Neuroimmunol..

[3] Scoville W.B., Milner B. (1957). Loss of recent memory after bilateral hippocampal lesions. J. Neurol. Neurosurg. Psychiatry.

[4] Schmitt F.A., Wichems C.H. (2006). A systematic review of assessment and treatment of moderate to severe Alzheimer’s disease. Prim. Care Companion J. Clin. Psychiatry.

[5] Surabhi, Singh B.K. (2019). Alzheimer’s disease: a comprehensive review. Int. J. Pharm. Sci. Res..

[6] Bhushan I., Kour M., Kour G., Gupta S. (2018). Alzheimer’s disease: causes & treatment – a review. Ann. Biotechnol..

[7] Weller J., Budson A. (2018). Review on Current understanding of Alzheimer’s disease diagnosis and treatment. F1000 Res..

[8] Thies, Bleiler (2013). Alzheimer’s disease facts and figures. Alzheimer Dement.

[9] Maity S., Nandy S., Mukherjee A., Dey A., Ullah M.F., Ahmad A. (2019). Nutraceuticals and Natural Product Derivatives: Disease Prevention & Drug Discovery.

[10] Meur S., Karati D. (2024). Fyn kinase in Alzheimer’s disease: unraveling molecular mechanisms and therapeutic implications. Mol. Neurobiol..

[11] Bullock R., Hammond G. (2003). Realistic expectations: the management of severe Alzheimer disease. Alzheimer Dis. Assoc. Disord..

[12] Bullock R. (2004). The needs of the caregiver in the long-term treatment of Alzheimer disease. Alzheimer Dis. Assoc. Disord..

[13] Bullock R. (2006). Efficacy and safety of memantine in moderate-to-severe Alzheimer disease: the evidence to date. Alzheimer Dis. Assoc. Disord..

[14] Meur S., Mukherjee S., Roy S., Karati D. (2024). Role of PIM kinase inhibitor in the treatment of Alzheimer’s disease. Mol. Neurobiol. 1-5.

[15] Karati D., Shaw T.K. (2022). Pharmacological importance of Bacopa monnieri on neurological disease (Alzheimer ’s disease) and diabetic neuropathy-a concise review. Res. J. Pharm. Technol. 15(8.

[16] Anand T., Naika M., Swamy M.S.L., Khanum F. (2011). Antioxidant and DNA damage preventive properties of *Bacopa monniera* (L) Wettst. Free Radic. Antioxid..

[17] Tripathi Y.B., Chaurasia S., Tripathi E., Upadhyay A., Dubey G.P. (1996). *Bacopa monniera* Linn. as an antioxidant: mechanism of action. Indian J. Exp. Biol..

[18] Bhattacharya S.K., Bhattacharya A., Kumar A., Ghosal S. (2000). Antioxidant activity of *Bacopa monniera* in rat frontal cortex, striatum and hippocampus. Phytother. Res.

[19] Sairam K., Dorababu M., Goel R.K., Bhattacharya S.K. (2002). Antidepressant activity of standardized extract of *Bacopa monniera*in experimental models of depression in rats. Phytomedicine.

[20] Sairam K., Rao C.V., Babu M.D., Goel R.K. (2001). Prophylactic and curative effects of *Bacopa monniera* in gastric ulcer models. Phytomedicine.

[21] Sumathy T., Govindasamy S., Balakrishna K., Veluchamy G. (2002). Protective role of *Bacopa monniera* on morphine-induced brain mitochondrial enzyme activity in rats. Fitoterapia.

[22] Yallappa B.S., Veeranna R.K., Suganthi R.U., Ravindra J., Amaladhas H.P., Vaibhav A., David C. (2021). Protective role of bacoside A on blood brain barrier integrity. Uttar Pradesh J. Zool..

[23] Calabrese V., Butterfield D.A., Stella A. (2003). Nutritional antioxidants and the heme oxygenase pathway of stress tolerance: novel targets for neuroprotection in Alzheimer’s disease. Ital. J. Biochem.

[24] Emilien G., Beyreuther K., Masters C.L., Maloteaux J.-M. (2000). Prospects for pharmacological intervention in Alzheimer disease. Arch. Neurol..

[25] Kontush A., Schekatolina S. (2004). Vitamin E in neurodegenerative disorders: Alzheimer’s disease. Ann. NY Acad. Sci..

[26] Maity S., Nandy S., Mukherjee A., Dey A., Ullah M.F., Ahmad A. (2019). Nutraceuticals and Natural Product Derivatives: Disease Prevention & Drug Discovery.

[27] Selkoe D.J. (2001). Alzheimer’s disease: genes, proteins, and therapy. Physiol. Rev..

[28] Brunkan A.L., Goate A.M. (2005). Presenilin function and gamma-secretase activity. J. Neurochem.

[29] Priller C., Bauer T., Mitteregger G., Krebs B., Kretzschmar H.A., Herms J. (2006). Synapse formation and function is modulated by the amyloid precursor protein. J. Neurosci..

[30] Cai Y., An S.S., Kim S. (2015). Mutations in presenilin 2 and its implications in Alzheimer’s disease and other dementia-associated disorders. Clin. Inter. Aging.

[31] Poirier J., Delisle M.C., Quirion R., Aubert I., Farlow M., Lahiri D. (1995). Apolipoprotein E4 allele as a predictor of cholinergic deficits and treatment outcome in Alzheimer disease. Proc. Natl. Acad. Sci. USA.

[32] Sinha M., Bhowmick P., Banerjee A., Chakrabarti S. (2013). Antioxidant role of amyloid β protein in cell-free and biological systems: implication for the pathogenesis of Alzheimerdisease. Free Radic. Biol. Med..

[33] Karati D., Meur S., Roy S., Mukherjee S., Debnath B., Jha S.K., Sarkar B.K., Naskar S., Ghosh P. (2024). Glycogen synthase kinase 3 (GSK3) inhibition: a potential therapeutic strategy for Alzheimer’s disease. Naunyn-Schmiede 'S. Arch. Pharmacol..

[34] Zhang Z., Yang X., Song Y.Q., Tu J. (2021 Dec 1). Autophagy in Alzheimer’s disease pathogenesis: therapeutic potential and future perspectives. Ageing Res. Rev..

[35] Sharma S., Rathi N., Kamal B. (2010). Conservation of biodiversity of highly important medicinal plants of India through tissue culture technology- a review. Agric. Biol. J. North Am..

[36] Roy A. (2017). A review on pharmaceutically important medicinal plant: *Bacopa monnieri*. J. Nat. Prod. Plant Resour..

[37] Vishnupriya P., Padma V.V. (2017). A review on the antioxidant and therapeutic potential of *Bacopa monnieri*. React. Oxyg. Spec..

[38] Murthy P.B.S. (2006). Estimation of twelve bacopa saponins in *Bacopa monnieri* extracts and formulations by high-performance liquid chromatography. Chem. Pharm. Bull..

[39] Deepak M. (2005). Quantitative determination of the major saponin mixture bacoside a in *Bacopa monnieri*by HPLC. Phytochem. Anal..

[40] Deepak M., Amit A. (2013). Bacoside B’ - the need remains for establishing identity. Fitoterapia.

[41] Le X.T., Nguyet P.H.T., Van N.T. (2015). Protective effects of *Bacopa monnieri* on ischemia induced cognitive deficits in mice: the possible contribution of bacopaside I and underlying mechanism. J. Ethno-Pharm..

[42] Jasim B., Daya P.S., Sreelakshmi K.S. (2017). Bacopaside N1 biosynthetic potential of endophytic Aspergillus sp. BmF 16 isolated from *Bacopa monnieri*. 3 Biotech.

[43] Christopher C., Johnson A.J., Mathew P.J., Baby S. (2017). Elite genotypes of *Bacopa monnieri*, with high contents of Bacoside A and Bacopaside I, from southern Western Ghats in India. Ind. Crops Prod..

[44] Srivastava P., Raut H.N., Puntambekar H.M., Desai A.C. (2012). Stability studies of crude plant material of *Bacopa monnieri* and quantitative determination of bacopaside I and bacoside A by HPLC. Phytochem Anal..

[45] Singh R., Ramakrishna R., Bhateria M., Bhatta R.S. (2014). In vitro evaluation of *Bacopa monniera* extract and individual constituents on human recombinant monoamine oxidase enzymes. Phytother. Res.

[46] Saitgareeva A.R., Bulygin K.V., Gareev I.F., Beylerli O.A., Akhmadeeva L.R. (2020). The role of microglia in the development of neurodegeneration. Neurol. Sci..

[47] Smith J.A., Das A., Ray S.K., Banik N.L. (2012). Role of pro-inflammatory cytokines released from microglia in neurodegenerative diseases. Brain Res. Bull..

[48] Zhang F., Jiang L. (2015). Neuroinflammation in Alzheimer’s disease. Neuropsychiatry Dis. Treat..

[49] Shih R.H., Wang C.Y., Yang C.M. (2015). NF-kappaB signaling pathways in neurological inflammation: a mini review. Front. Mol. Neurosci..

[50] Wang W.Y., Tan M.S., Yu J.T., Tan L. (2015). Role of pro-inflammatory cytokines released from microglia in Alzheimer’s disease. Ann. Transl. Med..

[51] Channa S., Dar A., Anjum S., Yaqoob M., Atta Ur R. (2006). Anti-inflammatory activity of Bacopa monniera in rodents. J. Ethnopharmacol..

[52] Nemetchek M.D., Stierle A.A., Stierle D.B., Lurie D.I. (2017). The Ayurvedic plant Bacopa monnieri inhibits inflammatory pathways in the brain. J. Ethnopharmacol..

[53] Williams R., Münch G., Gyengesi E., Bennett L. (2014). Bacopa monnieri (L.) exerts anti-inflammatory effects on cells of the innate immune system in vitro. Food Funct..

[54] Anwar S., Shamsi A., Shahbaaz M., Queen A., Khan P. (2020). Rosmarinic acid exhibits anticancer effects via MARK4 inhibition. Sci. Rep..

[55] Fatima U., Roy S., Ahmad S., Al-Keridis L.A., Alshammari N., Adnan M., Islam A., Hassan M.I. (2022). Investigating neuroprotective roles of Bacopa monnieri extracts: mechanistic insights and therapeutic implications. Biomed. Pharmacother..

[56] Sami N., Rahman S., Kumar V., Zaidi S., Islam A., Ali S., Ahmad F., Hassan M.I. (2017). Protein aggregation, misfolding and consequential human neurodegenerative diseases. Int. J. Neurosci..

[57] Kumar V., Sami N., Kashav T., Islam A., Ahmad F., Hassan M.I. (2016). Protein aggregation and neurodegenerative diseases: from theory to therapy. Eur. J. Med. Chem..

[58] Ternchoocheep K., Ingkaninan K., Yasothornsrikul S. (2012). Tau protein attenuation ability of Bacopa monnieri extract on nerve growth factordeprived PC12 cells in normal-serum and serum-free medium. Chiang Mai Med. J..

[59] Fatima U., Roy S., Ahmad S., Al-Keridis L.A., Alshammari N., Adnan M., Hassan M.I. (2022). Investigating neuroprotective roles of Bacopa monnieri extracts: mechanistic insights and therapeutic implications. Biomed. Pharmacother..

[60] Agarwal A., Mishra B., Gupta A., Srivastava M.V., Basheer A., Sharma J., Vishnu V.Y. (2023). Importance of high-quality evidence regarding the use of Bacopa monnieri in dementia. Front. Aging Neurosci..

[61] Mathew J., Soman S., Sadanandan J., Paulose C.S. (2010). Decreased GABA receptor in the striatum and spatial recognition memory deficit in epileptic rats: effect of Bacopa monnieri and bacoside-A. J. Ethnopharmacol..

[62] Banerjee M., Modi P. (2010). Micropropagation of Bacopa monnieri using cyanobacterial liquid medium. Plant Tissue Cult. Biotechnol..

[63] Antony Ceasar S., Lenin Maxwell S., Bhargav Prasad K., Karthigan M., Ignacimuthu S. (2010). Highly efficient shoot regeneration of Bacopa monnieri (L.) using a two-stage culture procedure and assessment of genetic integrity of micropropagated plants by RAPD. Acta Physiol. Plant..

[64] Saraf M.K., Prabhakar S., Khanduja K.L., Anand A. (2011). *Bacopa monniera* attenuates scopolamine-induced impairment of spatial memory in mice. Evid. Based Complement Altern. Med..

[65] Mukherjee S., Dugad S., Bhandare R., Pawar N., Jagtap S., Pawar P.K. (2011). Evaluation of comparative free-radical quenching potential of Brahmi (*Bacopa monnieri*) and Mandookparni (Centella asiatica). Ayu.

[66] Stough C.K., Pase M.P., Cropley V., Myers S., Nolidin K., King R. (2012). A randomized controlled trial investigating the effect of Pycnogenol and Bacopa CDRI08 herbal medicines on cognitive, cardiovascular, and biochemical functioning in cognitively healthy elderly people: the Australian Research Council Longevity Intervention (ARCLI) study protocol (ANZCTR12611000487910). Nutr. J..

[67] Chaudhari K.S., Tiwari N.R., Tiwari R.R., Sharma R.S. (2017). Neurocognitive effect of nootropic drug Brahmi (*Bacopa monnieri*) in Alzheimer’s disease. Ann. Neurosci..

[68] Sekhar V.C., Viswanathan G., Baby S. (2017). Insights into the molecular aspects of neuroprotective Bacoside A and Bacopaside I. Curr. Neuropharmacol..

[69] McQuiston A.R. (2014). Acetylcholine release and inhibitory interneuron activity in hippocampal CA1. Front Synaptic Neurosci..

[70] Orta-Salazar E., Cuellar-Lemus C.A., Díaz-Cintra S., Feria-Velasco A.I. (2014). Cholinergic markers in the cortex and hippocampus of some animal species and their correlation to Alzheimer's disease. Neurologia.

[71] Čolović M.B., Krstić D.Z., Lazarević-Pašti T.D., Bondžić A.M., Vasić V.M. (2013). Acetylcholinesterase inhibitors: pharmacology and toxicology. CurrNeuropharmacol.

[72] Holcomb L.A., Dhanasekaran M., Hitt A.R., Young K.A., Riggs M., Manyam B.V. (2006). *Bacopa monniera* extract reduces amyloid levels in PSAPP mice. J. Alzheimers Dis..

[73] Paulose C., Chathu F., Khan S., Krishnakumar A. (2008). Neuroprotective role of *Bacopa monnieri* extract in epilepsy and effect of glucose supplementation during hypoxia: glutamate receptor gene expression. Neurochem Res..

[74] McEwen B.S. (2004). Structural plasticity of the adult brain: how animal models help us understand brain changes in depression and systemic disorders related to depression. Dialog-. Clin. Neurosci..

[75] Zheng F., Luo Y., Wang H. (2009). Regulation of brain derived neurotrophic factor-mediated transcription of the immediate early gene Arc by intracellular calcium and calmodulin. J. Neurosci. Res..

[76] Drozdov O., Chorna V. (2003). Changes in the content of glial fibrillary acidic protein in the frontal cortex of rats during conditioned active avoidance training. Neurophysiology.

[77] Konar A., Shah N., Singh R., Saxena N., Kaul S.C., Wadhwa R. (2011). Protective role of Ashwagandha leaf extract and its component withanone on scopolamine-induced changes in the brain and brain-derived cells. PLoS One.

[78] Masliah E., Mallory M., Deerinck T., DeTeresa R., Lamont S., Miller A. (1993). Re-evaluation of the structural organization of neuritic plaques in Alzheimer’s disease. J. Neuropathol. Exp. Neurol..

[79] Chaudhari K.S., Tiwari N.R., Tiwari R.R., Sharma R.S. (2017). Neurocognitive effect of nootropic drug Brahmi (Bacopa monnieri) in Alzheimer's disease. Ann. Neurosci..

[80] Mathew M., Subramanian S. (2012). Evaluation of the anti-amyloidogenic potential of nootropic herbal extracts in vitro. Int. J. Pharm. Sci. Res..

[81] Holcomb L.A., Dhanasekaran M., Hitt A.R., Young K.A., Riggs M., Manyam B.V. (2006). Bacopa monniera extract reduces amyloid levels in PSAPP mice. J. Alzheimers Dis..

[82] Limpeanchob N., Jaipan S., Rattanakaruna S., Phrompittayarat W., Ingkaninan K. (2008). Neuroprotective effect of Bacopa monnieri on beta-amyloid-induced cell death in primary cortical culture. J. Ethnopharmacol..

[83] Limpeanchob N., Jaipan S., Rattanakaruna S., Phrompittayarat W., Ingkaninan K. (2008). Neuroprotective effect of Bacopa monnieri on beta-amyloid-induced cell death in primary cortical culture. J. Ethnopharmacol..

[84] Pandareesh M., Anand T. (2013). Neuromodulatory propensity of Bacopa monniera against scopolamine-induced cytotoxicity in PC12 cells via down-regulation of AChE and up-regulation of BDNF and muscarnic-1 receptor expression. Cell Mol. Neurobiol..

[85] Malishev R., Shaham-Niv S., Nandi S., Kolusheva S., Gazit E., Jelinek R. (2017). Bacoside-A, an Indian traditional-medicine substance, inhibits β-amyloid cytotoxicity, fibrillation, and membrane interactions. ACS Chem. Neurosci..

[86] Aithal M.G., Rajeswari N. (2019). Bacoside A induced sub-G0 arrest and early apoptosis in human glioblastoma cell line U-87 MG through notch signaling pathway. Brain Tumor Res. Treat..

[87] Singh B., Pandey S., Rumman M., Kumar S., Kushwaha P.P., Verma R. (2021). Neuroprotective and neurorescue mode of action of Bacopa monnieri (L.) Wettst in 1-methyl-4-phenyl-1, 2, 3, 6-tetrahydropyridine-induced Parkinson's disease: an in silico and in vivo study. Front Pharm..

[88] Piyabhan P., Tingpej P., Duansak N. (2019). Effect of pre-and post-treatment with Bacopa monnieri (Brahmi) on phencyclidine-induced disruptions in object recognition memory and cerebral calbindin, parvalbumin, and calretinin immunoreactivity in rats. Neuropsychiatr. Dis. Treat..

[89] Kunte K.B., Kuna Y. (2013). Neuroprotective effect of Bacopa monniera on memory deficits and ATPase system in Alzheimer's disease (AD) induced mice. J. Sci. Innov. Res.

[90] Aluko R.E. (2021). Food-derived Acetylcholinesterase Inhibitors as Potential Agents against Alzheimer’s Disease. Efood.

[91] Jeyasri R., Muthuramalingam P., Suba V., Ramesh M., Chen J.T. (2020). Bacopa monnieri and their bioactive compounds inferred multi-target treatment strategy for neurological diseases: a cheminformatics and system pharmacology approach. Biomolecules.

[92] Shoukat S., Zia M.A., Uzair M., Attia K.A., Abushady A.M., Fiaz S., Ali G.M. (2023). Bacopa monnieri: a promising herbal approach for neurodegenerative disease treatment supported by in silico and in vitro research. Heliyon.

[93] Roy S., Chakravarty S., Talukdar P., Talapatra S.N. (2019). Identification of bioactive compounds present in Bacopa monnieri Linn. Against caspase-3 and tau protein kinase I to prevent Alzheimer’s disease: an in-silico study. Pharma Innov. J..

